# Assessing cardiovascular stress based on heart rate variability in female shift workers: a multiscale-multifractal analysis approach

**DOI:** 10.3389/fnrgo.2024.1382919

**Published:** 2024-05-09

**Authors:** Raquel Delgado-Aranda, Guadalupe Dorantes-Méndez, Anna Maria Bianchi, Juha M. Kortelainen, Stefania Coelli, Jorge Jimenez-Cruz, Martin O. Méndez

**Affiliations:** ^1^Facultad de Ciencias, Universidad Autónoma de San Luis Potosí, San Luis Potosí, Mexico; ^2^Department of Electronics, Information and Bioengineering, Politecnico di Milano, Milano, Italy; ^3^VTT Technical Research Center of Finland, Tampere, Finland; ^4^Department of Obstetrics and Prenatal Medicine, University Hospital Bonn, Bonn, Germany

**Keywords:** cardiovascular stress, detrended fluctuation analysis, heart rate variability, shift work, sleep

## Abstract

**Introduction:**

Sleep-wake cycle disruption caused by shift work may lead to cardiovascular stress, which is observed as an alteration in the behavior of heart rate variability (HRV). In particular, HRV exhibits complex patterns over different time scales that help to understand the regulatory mechanisms of the autonomic nervous system, and changes in the fractality of HRV may be associated with pathological conditions, including cardiovascular disease, diabetes, or even psychological stress. The main purpose of this study is to evaluate the multifractal-multiscale structure of HRV during sleep in healthy shift and non-shift workers to identify conditions of cardiovascular stress that may be associated with shift work.

**Methods:**

The whole-sleep HRV signal was analyzed from female participants: eleven healthy shift workers and seven non-shift workers. The HRV signal was decomposed into intrinsic mode functions (IMFs) using the empirical mode decomposition method, and then the IMFs were analyzed using the multiscale-multifractal detrended fluctuation analysis (MMF-DFA) method. The MMF-DFA was applied to estimate the self-similarity coefficients, α(*q*, τ), considering moment orders (*q*) between –5 and +5 and scales (τ) between 8 and 2,048 s. Additionally, to describe the multifractality at each τ in a simple way, a multifractal index, *MFI*(τ), was computed.

**Results:**

Compared to non-shift workers, shift workers presented an increase in the scaling exponent, α(*q*, τ), at short scales (τ < 64 s) with *q* < 0 in the high-frequency component (IMF1, 0.15–0.4 Hz) and low-frequency components (IMF2–IMF3, 0.04–0.15 Hz), and with *q*> 0 in the very low frequencies (IMF4, < 0.04 Hz). In addition, at large scales (τ> 1,024 s), a decrease in α(*q*, τ) was observed in IMF3, suggesting an alteration in the multifractal dynamic. *MFI*(τ) showed an increase at small scales and a decrease at large scales in IMFs of shift workers.

**Conclusion:**

This study helps to recognize the multifractality of HRV during sleep, beyond simply looking at indices based on means and variances. This analysis helps to identify that shift workers show alterations in fractal properties, mainly on short scales. These findings suggest a disturbance in the autonomic nervous system induced by the cardiovascular stress of shift work.

## 1 Introduction

Shift work is common in various sectors, including industry and clinical areas, due to increased demand for services and products. Shift work involves working in various shifts or outside of morning schedules (Puttonen et al., 2010). However, long working hours and rotating shifts can induce occupational and physiological stress, potentially elevating the long-term risk of cardiovascular disease (Puttonen et al., 2010; Kivimäki and Steptoe, 2018).

Shift workers experience a circadian misalignment because sleep schedules are modified due to night shifts. The disruption of circadian rhythms and sleep disorders caused by shift work generate cardiovascular stress that negatively impacts health. Shift work has been associated with a higher risk of cardiovascular diseases and ischemic strokes (James et al., 2017). Additionally, compared to non-shift workers, there is an increased risk of coronary heart disease due to the chronic stress associated with shift work (Tenkanen et al., 1997).

Several studies have evaluated the impact of shift work on the cardiovascular system. Assessment of the cardiovascular system is usually performed by evaluating the characteristics of the inter-beat interval (IBI) calculated from the electrocardiogram (ECG), better known as heart rate variability (HRV) analysis. Specific time and frequency indices obtained from the IBI signal, such as high-frequency (HF) and low-frequency (LF), are directly related to the sympathetic and parasympathetic branches of the autonomic nervous system (ANS). Therefore, it is possible to evaluate the behavior of the cardiovascular system non-invasively.

The alterations in the cardiovascular system for shift working have been studied by considering different regimes such as permanent or rotating shift work (Chung et al., 2011; Hulsegge et al., 2018), data acquisition during wakefulness (Kunikullaya et al., 2010; Hsu et al., 2021), sleep (Chung et al., 2011; Hulsegge et al., 2018), pre- and post-shift (Shen et al., 2016), 24h acquisitions (Lee et al., 2015), or during cardiovascular stress test as postural change (Monteze et al., 2015). These studies have reported that shift workers during wakefulness present an increase in low to high frequency ratio (LF/HF) and low-frequency power as absolute and normalized values (Hsu et al., 2021). Instead, during sleep, shift workers showed an increase in sympathetic activity (Neufeld et al., 2017) and a decrease in very-low-frequency (VLF) (Hulsegge et al., 2018). In addition, Munakata et al. (2001) reported higher HF during night work with respect to day work. van Amelsvoort et al. (2001a) described an increase in the frequency of extrasystoles in shift workers without other HRV differences. Other studies suggest that long-standing shift work is associated with decreased parasympathetic modulation and increased blood pressure, promoting a gradual worsening of cardiovascular health status (Souza et al., 2015). Conversely, other authors have reported no significant differences in temporal and spectral IBI indices between non-shift workers and shift workers (Kunikullaya et al., 2010; Chung et al., 2012). Therefore, it will be useful to further extend the HRV analysis to better understand the changes caused by shift work.

As discussed above, studies have been focused on the time and frequency domain of IBI, as it is a well-established tool and provides a quantitative assessment of sympathetic and parasympathetic nervous system activation. However, the property of self-similarity is an intrinsic feature of the IBI signal and so far has not been used to characterize IBI behavior in shift workers during sleep. The self-similarity property means that a small part of a time series exhibits statistical properties related to a larger part of the time series (Hardstone et al., 2012), and it could be measured by the degree of persistence in which the values of a time series are correlated (Kantelhardt, 2012).

There are different methods for assessing the self-similarity in biological signals, for example, autocorrelation function analysis, spectral analysis, fluctuation analysis, and Hurst's rescaled-range analysis (Castiglioni et al., 2017). However, the beat-by-beat dynamics of several cardiovascular signals during sleep present non-stationary and non-linear behavior, which are observed in the different sleep stages. To cope with these problems, an extension of the Fluctuation Analysis, called Detrended Fluctuation Analysis (DFA), has been proposed (Peng et al., 1994) and successfully applied to a wide range of physiological time series. Initially, two ranges of scales were considered to evaluate the self-similarity of HRV (Peng et al., 1995). However, changes in self-similarity of heart rate were observed as the scale increased (Castiglioni et al., 2009), and DFA has been adapted to evaluate the multiscale-multifractal characteristics of the HRV (Castiglioni and Faini, 2019).

The high and low-frequency components of HRV show remarkable variations during the different cyclical sleep stages. The joint interaction of these components leads to fractal dynamics in cardiac activity, frequently characterizing as self-similarity with 1/f behavior. However, to our knowledge, the multiscale multifractal structure of the high and low-frequency components separately has not been evaluated, which may reveal significant information about HRV during sleep. Moreover, since the high frequency component predominates during sleep, its separate evaluation could put into evidence important aspects of HRV dynamics. Furthermore, this analysis has the potential to improve the understanding and characterization of changes in cardiac stress induced by shift work from a multifractal perspective.

Assessing the impact of shift work stress through HRV is crucial due to its association with an increased risk of cardiovascular disease (Puttonen et al., 2010). Using a multiscale-multifractal approach to evaluate HRV components enables the characterization of changes in HRV properties due to shift work and the identification of the components that undergo the greatest alterations. In addition, understanding changes in HRV related to cardiovascular stress in shift workers could have important implications for the early detection of potential cardiovascular health problems. This could lead to preventive interventions to improve the long-term health and well-being of these workers.

The present study aims to compare the condition of the cardiovascular system of shift workers and non-shift workers women by IBI signal analysis, to better understand the effect of physiological stress produced by shift work. Given that the IBI signal can be decomposed into fast and slow waves, we also propose to analyze the multiscale-multifractal dynamics of these different components. The self-similarity of IBI spectral components was analyzed using MMF-DFA.

## 2 Materials and methods

The IBI signal during sleep was analyzed using a multiscale-multifractal approach in women with shift and non-shift work schedules. In addition, it was proposed to analyze the fractal proprieties of the high and low-frequency components of the IBI signal. HRV analysis was carried out using Matlab^®^ R2018a (The MathWorks, Inc., Natick, Massachusetts, United States).

### 2.1 Database

Group 1. Sleep recordings of 11 healthy female shift workers (age 20–54 years) were acquired in the sleep laboratory of the Finnish Institute of Occupational Health. The participants did not present any neurological disorders and were free of drugs affecting the central nervous system. The study was approved by the local Ethics Committee, and the participants gave their written informed consent to participate. Daytime and nighttime sleep were acquired; however, only nighttime sleep recordings were analyzed in this work. All participants underwent full polysomnography and pressure bed sensor (PBS) recordings were performed simultaneously. The PBS was designed with eight sensors to measure the pressure change under the torso of the sleeping participant. The sensors were placed in two columns and four rows, covering an area of 64 cm × 64 cm. The sampling rate of the sensors was 50 Hz for each measurement channel. Ballistocardiography (BCG), respiration, and movement signals were extracted from the PBS signal by filtering procedures (Guerrero et al., 2015). The signal processing was based on the IBI signal obtained from BCG overnight, and from this signal, the HRV analysis was performed. The comparison of the IBI signals extracted from BCG or ECG has shown that both signals give similar results in HRV analysis (Kortelainen et al., 2010).

Group 2. ECG signals from seven healthy women (age 25–35 years) were analyzed from the CAP Sleep database (Goldberger et al., 2000, 2012). The full polysomnographic recordings of the CAP Sleep Database were registered at the Sleep Disorders Center of the Ospedale Maggiore of Parma, Italy. Sampling frequencies of ECG signals varied between 100 and 512 Hz, and the RR intervals were detected according to Pan and Tompkins (1985). The women participants did not present any neurological disorders and were free of drugs affecting the central nervous system. This group will be called non-shift workers.

In the following sections, IBI is used to refer to IBI and RR interval time series. The IBI signal was manually reviewed and pre-processed with an adaptive filter to remove artifacts and ectopic beats in both groups (Wessel et al., 2000).

### 2.2 Decomposition of the IBI signal

The complete ensemble empirical mode decomposition with adaptive noise (CEEMDAN) is based on the empirical mode decomposition (EMD) method. The EMD method decomposes the signal *x* into *K* intrinsic mode functions (IMFs, *d*_*i*_) that, together with a residue (*r*_*k*_) reconstruct the signal as $x=\overset{K}{\underset{i=0}{\sum }}d_{i}+r_{k}$ (Huang et al., 1998). An IMF must satisfy the conditions that the number of extrema and the number of zero-crossings must be equal or differ by one at most, and the local mean, defined as the mean of the upper and lower envelopes, must be zero (Huang et al., 1998). Let *E*_*k*_(·) be the operator that produces the *k*th mode and let ω^(*i*)^ be a realization of zero mean unit variance white noise. According to Torres et al. (2011) and Colominas et al. (2014), the CEEMDAN method consists of the following steps:

1. Decompose by EMD each $x^{\left(i\right)}=x+\beta_{0}\omega^{\left(i\right)}$, *i* = 1, …, *I*, until its first mode and calculate $\tilde{d_{1}}=\frac{1}{I}\overset{I}{\underset{i=1}{\sum }}d_{1}^{\left(i\right)}$.
2. For *k* = 1, obtain the first residue as $r_{1}=x-\tilde{d_{1}}$.
3. Obtain the first mode of $r_{1}+\beta_{1}E_{1}\left(\omega^{\left(i\right)}\right),\;i=1,\ldots ,I$, by EMD and define the second mode as $\tilde{d_{2}}=\frac{1}{I}\overset{I}{\underset{i=1}{\sum }}E_{1}\left(r_{1}+\beta_{1}E_{1}\left(\omega^{\left(i\right)}\right)\right)$.
4. For *k* = 2, …, *K* calculate the *k*th residue as $r_{k}=r_{\left(k-1\right)}-\tilde{d_{k}}$.
5. Obtain the first mode of $r_{k}+\beta_{k}E_{k}\left(\omega^{\left(i\right)}\right)$, *i* = 1, …, *I*, by EMD until define the (*k*+1)th CEEMDAN mode as ${\tilde{d}}_{k+1}=\frac{1}{I}\overset{I}{\underset{i=1}{\sum }}E_{1}\left(r_{k}+\beta_{k}E_{k}\left(\omega^{\left(i\right)}\right)\right)$.
6. Go to step 4 for the next *k*.

Repeat steps 4–6 until the EMD can not decompose the residue obtained. The final residue satisfies $r_{k}=x-\overset{K}{\underset{k=1}{\sum }}\tilde{d_{k}}$. Therefore, *x* can be expressed as $x=\overset{K}{\underset{k=1}{\sum }}\tilde{d_{k}}+r_{k}$.

**Figure 1:**
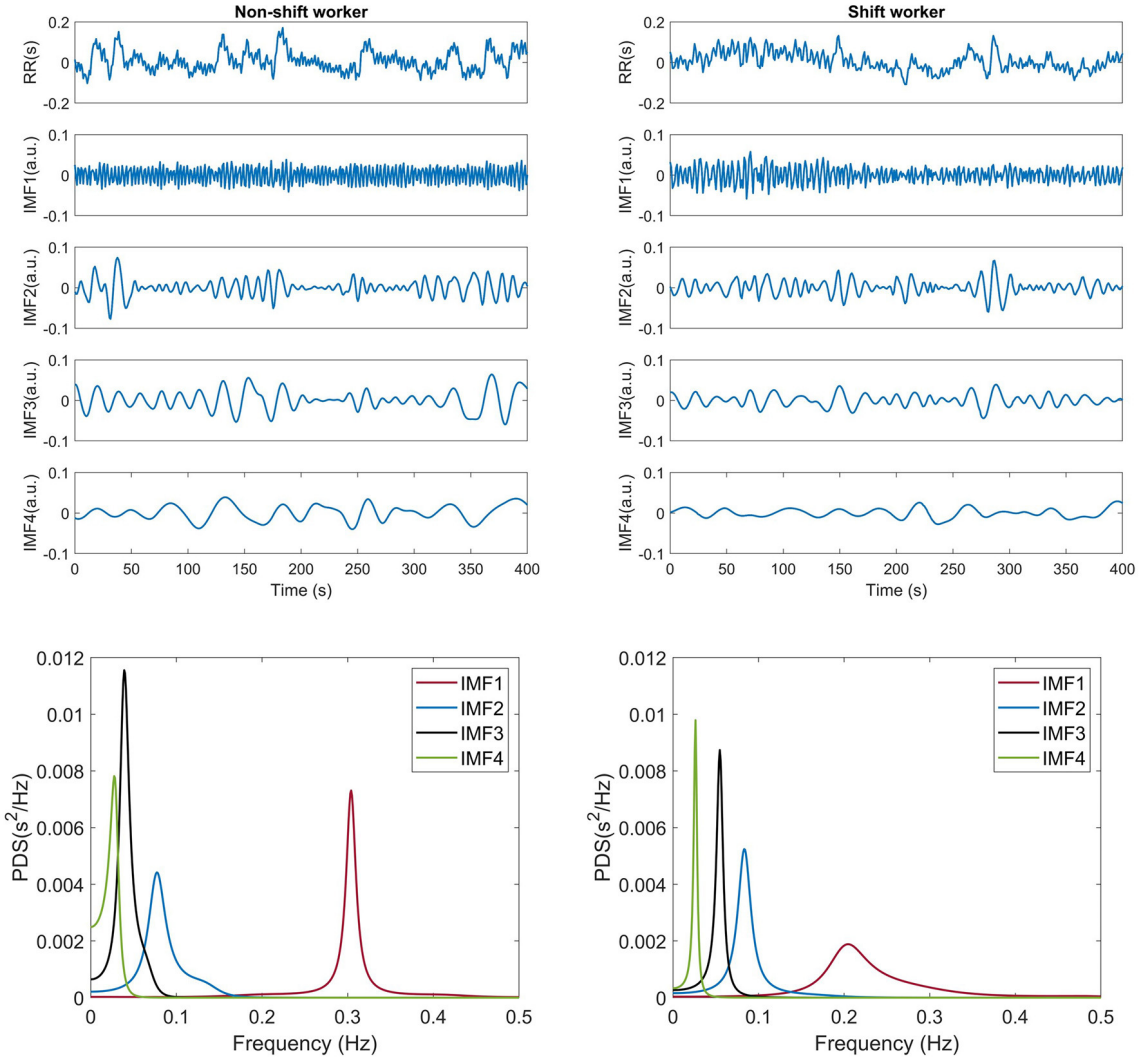
Example of decomposition of a 500-sample long RR interval using CEEMDAN and its frequency decomposition from a shift and non-shift workers.

For each participant, a segment of 9,000 heartbeats, ~2 h connected to the first sleep NREM-REM cycle, was extracted. Subsequently, the IBI signal was computed and decomposed into IMFs by CEEMDAN. An ensemble size of *I*= 100 was used and a value of 0.1 effectively reduced the residual noise in the reconstructed signal. Figure 1 presents an example of the signal decomposition of the RR interval signal of a non-shift worker (left panel) and a shift worker (right panel). Both participants showed an IMF1 that contained fast oscillations, while the following IMFs exhibited a decrease in oscillations. Consequently, IMF4 displayed slower oscillations. Therefore, frequency spectral analysis revealed distinct characteristics for each IMF, as illustrated in Figure 1. In both cases, IMF1 exhibited frequencies mainly falling between 0.15–0.4 Hz, IMF2 displayed frequencies approximately within the range of 0.04–0.2 Hz, IMF3 conveyed information mostly in the range of 0.04–0.15 Hz, and IMF4 featured frequencies primarily below 0.04 Hz. The IMF1–IMF4 was analyzed using the MMF-DFA described in the following section.

### 2.3 Multiscale-multifractal analysis

The MMF-DFA was calculated using the fast DFA algorithm proposed in Castiglioni and Faini (2019). A time series, *X*_*i*_ of *N* samples (*i* = 1, ..., *N*) is normalized as *x*_*i*_ = (*X*_*i*_−μ)/σ, where μ and σ are the mean and standard deviation of the series, respectively. Subsequently, the cumulative sum, *y*, is calculated. Then, *y* is divided into *M* maximally overlapped blocks of *n* samples. The trend of each block is removed with a polynomial least squares regression of order *p*, and the variance $\sigma_{n}^{2}$ of each detrended block *k* is calculated. Finally, the variability function *F*_*q*_(*n*) is obtained as


(1) $$\begin{array}{c} \;F_{q}(n)={\left\{\frac{1}{M}\sum_{k=1}^{M}{\left(\sigma_{n}^{2}(k)\right)}^{q/2}\right\}}^{1/q}\text{ for }q\neq 0\\ F_{q}(n)=\exp\left\{\frac{1}{2M}\sum_{k=1}^{M}\ln(\sigma_{n}^{2}(k))\right\}\text{ for }q=0 \end{array}$$

Equation (1) was evaluated for *q* between –5 and 5 and *n* between 6 and 2,048 to ensure at least 4 blocks in the computing of *F*_*q*_(*n*). For *q*>0, large fluctuations in the signal are analyzed, while, for *q* < 0, small fluctuations are analyzed. It has been reported that overestimation errors may affect shortest scales and negative *q*. To correct these errors, variances below an EPS threshold equal to 0.01 were not considered. The scales *n* were selected with a distribution evenly spaced on a logarithmic scale. Moreover, multiscale-multifractal coefficients, α(*q, n*), were evaluated as a function of scale *n* by calculating the derivative of log(*F*_*q*_(*n*)) vs. log(*n*) (Castiglioni and Faini, 2019).

Polynomials of orders 1 and 2 were used for detrending because it has been observed that there is overfitting with the second-order polynomial at scales smaller than 12 samples; however, it efficiently eliminates the long-term trend (Castiglioni and Faini, 2019). The *F*_*q*_(*n*) is presented in time scale by converting the *n* scale (samples) to τ scales (seconds) using the transformation τ = *n*×μ (Castiglioni and Faini, 2019). The MMF-DFA was computed for IMF1, IMF2, IMF3 and IMF4.

In addition, for each temporal scale τ, multifractality was quantified based on the standard deviation of α(*q*, τ) over the range of *q*, denoted as *MFI*(τ). The presence of multifractality implies that α(*q*, τ) depends on *q*. Therefore, if there is variability in α(*q*, τ) values across the range of *q*, *MFI*(τ) differs from zero, indicating multifractality at τ. Conversely, if α(*q*, τ) has the same or similar values, *MFI*(τ) takes values close to zero, indicating monofractal behavior at τ. Another index has been proposed that uses the range of *q* as a normalization factor (Castiglioni et al., 2018).

### 2.4 Statistical analysis

For each participant, the multiscale-multifractal self-similarity of a 9,000 heartbeat segment of the IBI signal during the first hours of sleep was examined. The Lilliefors test (Yap and Sim, 2011) is a statistical tool used to determine whether a sample comes from a normal distribution, and it was used to determine whether the multiscale-multifractal self-similarity for each group was normally distributed. Subsequently and in accordance with the previous test, the one-way ANOVA (Sheats and Pankratz, 2002) was used with *p* < 0.05 to determine significant differences. ANOVA is a method for comparing the means of several populations if they are approximately normally distributed. It is important to note, the α(*q*, τ) coefficients were compared between groups at each τ and *q*; regardless of the neighborhood.

## 3 Results

The results of the multifractal analysis performed on the HRV of women during the first hours of sleep, both under shift and non-shift conditions, are presented in this section. In the first subsection, we present the MMF-DFA coefficients, denoted as α(*q*, τ), for each respective group, accompanied by a detailed statistical comparison between the groups. Then, in the second subsection, the results of the Multifractal Index for each group will be discussed in detail, together with statistical comparisons between the groups.

The heart rate and the total power of the four IMFs were calculated, as shown in Table 1. The total power of each IMF was normalized with respect to the total power of the IBI signal. Shift workers showed a heart rate lower than non-shift workers (*p*-value < 0.05). However, the total power of each IMF was similar in both groups.

**Table 1:** Mean ± standard deviation of heart rate and total power of each IMF.

	**Non-shift workers**	**Shift workers**	***P*-value**
Heart rate (beats/min)	67.36 ± 5.28	58.76 ± 6.59	0.007^*^
IMF1 total power (a.u.)	0.14 ± 0.08	0.25 ± 0.10	0.26
IMF2 total power (a.u.)	0.08 ± 0.03	0.11 ± 0.06	0.32
IMF3 total power (a.u.)	0.15 ± 0.05	0.12 ± 0.04	0.16
IMF4 total power (a.u.)	0.14 ± 0.03	0.14 ± 0.05	0.89

^*^ *p* < 0.05 non-shift workers vs. shift workers.

### 3.1 MMF-DFA coefficients

**Figure 2:**
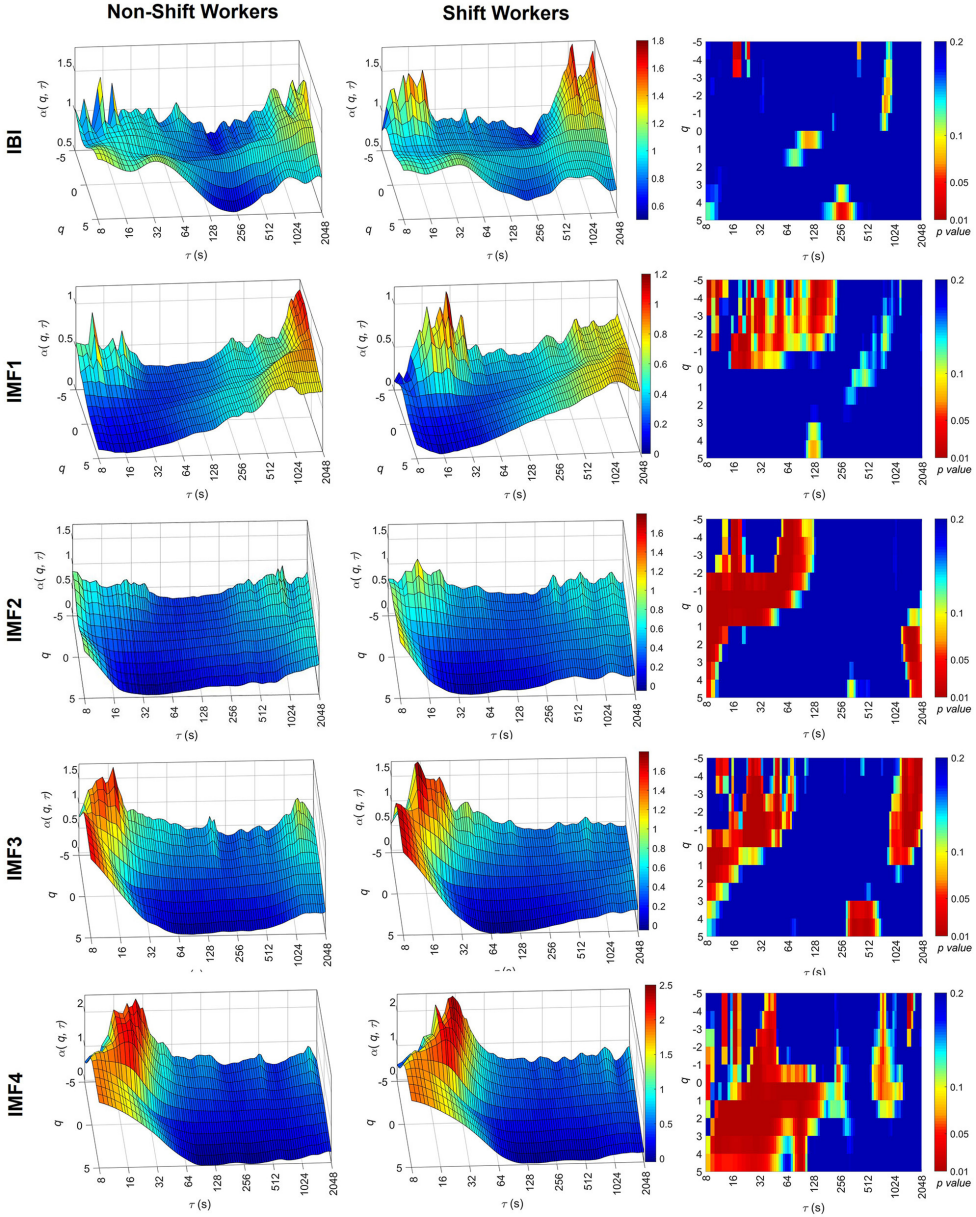
Multiscale-multifractal analysis of the IBI signal components. Average surfaces of MMF-DFA coefficients, α(*q*, τ), for non-shift workers **(left)** and shift workers **(center)**. Statistical significance of the MMF-DFA coefficients between both groups **(right)**. The surfaces of the coefficients are presented with different ranges on the α(*q*, τ) axis to facilitate the identification of differences between groups.

Figure 2 shows the averaged results of α(*q*, τ) for the IBI signal, along with the associated IMFs calculated for both non-shift and shift workers. Within the context of the IBI signal, the scaling coefficients consistently remain around 0.8 across different values of τ and *q*, indicating the monofractal property of IBI. Noticeable statistical differences were observed only in isolated regions, therefore, it remains difficult to draw definitive conclusions regarding the statistical differences between the groups.

IMF1 correlates predominantly with fast oscillations, specifically with frequencies between 0.15 Hz and 0.4 Hz. The α(*q*, τ) values are around 0.5, suggesting a white noise process. However, within scales spanning from 16 to 64, the shift working group manifests higher values in α(*q*, τ) for *q* negatives. These values of α(*q*, τ) in shift workers at short scales present statistical differences with respect to the non-shift groups (*p*-value < 0.05) and suggest a tendency of IMF1 to 1/f noise only for small scales.

IMF2 and IMF3 are related to the low frequency of the HRV, this means, with frequencies between 0.04 and 0.15 Hz. It is observed that both groups present a clear increment for α(*q*, τ) of scales lesser than 64 s, this values override the value of one. This suggests that small-scale time tends to present properties similar to the 1/f noise. However, when α(*q*, τ) is compared between groups, the shift workers present higher values than non-shift workers. This high value of α(*q*, τ) in shift workers at short scales was statically significant (*p*-value < 0.05), considering negative moments of *q*.

Finally, the results of the IMF4 show higher values in α(*q*, τ) were observed in τ < 128 s, mainly in the positive moments *q* of shift workers compared to non-shift workers.

### 3.2 Multifractal index

**Figure 3:**
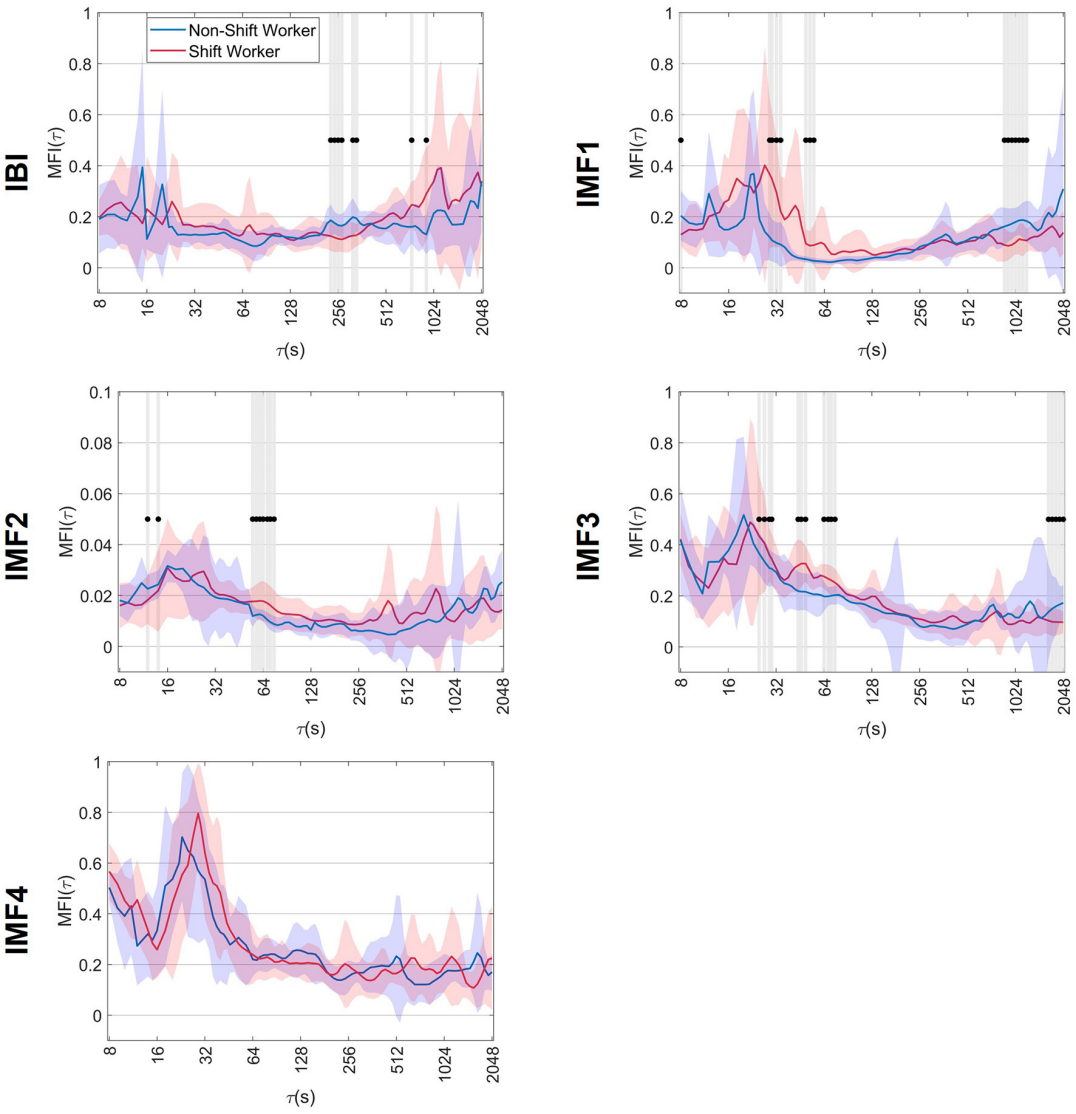
Mean ± standard deviation of the multifractal index, *MFI*(τ), of non-shift workers and shift workers. The symbol •, together with shaded bands, indicates the scales where significant differences vs. non-shift workers (*p* < 0.05).

Figure 3 shows the averaged *MFI*(τ) results for the IBI signal, along with the associated IMFs, for both non-shift and shift workers. The behavior of the *MFI*(τ) along the scales is represented by a solid blue and red line for non-shift workers and shift workers, respectively. In addition, the standard deviation of the index is represented by shading around the average line in blue and red, depending on the group. Also, scales where the *MFI*(τ) was significantly different between non-shift and shift workers are indicated by dots accompanied by shaded bands. In both groups, the *MFI*(τ) of the IBI signal had values close to 0.2 across different values of τ. On the other hand, the evaluation of the *MFI*(τ) in the IMFs reflected an increase of α(*q*, τ) variability at short scales, with values > 0.2, and a decrease of *MFI*(τ) at large scales, indicating a monofractal behavior. Also, statistical differences were observed between shift and non-shift workers at different τ scales, indicated by dots.

In the context of the IBI signal, a decrease in *MFI*(τ) was observed at scales close to 256 s in shift workers. These *MFI*(τ) values in shift workers show statistical differences compared to the non-shift workers (*p*-value < 0.05), suggesting a reduction in α(*q*, τ) variability at time scales near 256 s.

The multifractality quantion in IMF1 showed that shift workers had higher *MFI*(τ) values at 29 s ≤ τ ≤ 64 s scales. This increase in *MFI*(τ) at these scales was significantly different (*p*-value < 0.05) compared to non-shift workers, suggesting more variations of scaling exponents along *q*. In addition, a decrease in *MFI*(τ) was observed in shift workers at large scales, particularly near 1,024 s, which was statistically different (*p*-value < 0.05) from the values obtained in non-shift workers.

When analyzing the IMF2, it was observed that shift workers presented higher *MFI*(τ) values along all time scales compared to the values obtained in non-shift workers. This increase in *MFI*(τ) observed in shift workers was statistically significant (*p*-value < 0.05), particularly in the range of 55–75 s, suggesting a higher multifractal complexity of the IMF2 dynamics.

The IMF3 analysis showed that in both groups, *MFI*(τ) presented values > 0.2 in short scales and decreased as the τ scale increased. In addition, shift workers exhibited an increase in *MFI*(τ) on scales from 25 to 80 s and a decrease on scales > 1,024 s; these changes were significantly different (*p*-value < 0.05) compared to non-shift workers. Finally, despite the changes observed in the scaling exponents in IMF4, the *MFI*(τ) showed similar behavior in both groups, with no significant differences.

## 4 Discussion

In the present study, the self-similarity property of the IBI signal during sleep was evaluated using the MMF-DFA method in female shift and non-shift workers. To our knowledge, this is the first study to address changes in the multiscale-multifractal dynamics of HRV in shift workers. In addition, the analysis of the multiscale-multifractal dynamics of the fast and slow components of the IBI signal (obtained by the CEEMDAN method) was performed. Our main observations are: (a) In both groups, the IMFs presented multifractality at small scales, i.e., the multifractal index showed an increase at small scales, and (b) when multifractality is observed, it is different between shift workers and non-shift workers.

Previous studies have shown that the MMF-DFA method adequately describes the dynamics of the cardiovascular series during wakefulness and sleep under normal and pathological conditions (Kokosińska et al., 2018; Castiglioni et al., 2020); however, under shift work has not been evaluated. Many efforts have been performed regarding the analysis of HRV using time and frequency domain indices, where the findings are not conclusive, the results include higher LF of permanent night shift workers in comparison with rotating shift workers during daytime sleep (Chung et al., 2011), normalized LF during sleep significantly elevated on the night shift vs. the day shift (van Amelsvoort et al., 2001b), in contrast with a reduction in normalized LF and LF/HF during night work compared with other diurnal working periods (Furlan et al., 2000), increased HF during the night shift than during the day shift (Munakata et al., 2001), among other results. Therefore, the analysis of multifractal and multiscale characteristics of IBI signal can provide more insight in the ANS regulation changes in shift workers.

Moreover, previous efforts have evaluated the self-similarity of parasympathetic and sympathetic activity separately by administering autonomic blocking agents (Castiglioni et al., 2011; Moghtadaei et al., 2022). In contrast to Castiglioni et al. (2011), our study included the multifractal part by evaluating positive and negative moments (−5 ≤ *q* ≤ +5), analyzed longer signals during sleep (~2 h), and evaluated the high and low frequencies separately by decomposing the IBI signal using CEEMDAN.

In our initial analysis, we evaluated the α(*q*, τ) coefficients of the IBI signal among both shift workers and non-shift workers. The results showed a similar behavior of the scaling exponents; in fact, no statistically significant differences emerged between the two groups. In contrast, when the multiscale-multifractal behavior of the fast and slow wave components are evaluated separately, the scaling coefficients show significant changes at different scales and moments, suggesting a change in the ANS due to shift work. Therefore, it is evident that a multifractal analysis of the IBI signal is not sufficient to reveal the multifractal properties of the ANS, since the changes due to the interaction between the ANS branches maintain the monofractal structure of the IBI, which is independent of scale and *q*.

In the frequency domain of HRV analysis, HF is associated with parasympathetic nervous system activity. Given that IMF1 contains frequency information similar to HF, IMF1 could be associated with the dynamics of parasympathetic modulation. As illustrated in Figure 2, shift workers exhibit lower α(*q*, τ) values at large scales and higher values at short scales with *q* < 0. Consequently, these findings suggest that shift workers present an alteration in the dynamics of parasympathetic modulation. Changes in parasympathetic activity were also observed in Hulsegge et al. (2018), where the relationship between shift work and cardiac activity during sleep was evaluated, considering differences by age and sex. The results from that study suggested that compared to non-shift working women, shift-working women have an autonomic balance in favor of parasympathetic activity.

IMF2 and IMF3 could also be associated with sympathetic modulation, as they contain information in the 0.04–0.15 Hz range, corresponding to LF of HRV spectral analysis. Our results of IMF3 in non-shift workers coincide with the analysis performed in Castiglioni et al. (2018), where it is suggested that at short scales, the process modulating sympathetic heart rate activity is similar to fractional Brownian motion, and at large scales, it is similar to fractional Gaussian noise. The behavior described above is also observed in shift workers; however, α(*q*, τ) is significantly larger at short scales. In contrast, at large scales, shift workers exhibit a decrease in *MFI*(τ), suggesting less variability in the scaling exponents and a decrease in fractality, and an alteration in the modulation of sympathetic activity. These changes in sympathetic modulation could be associated with the increased cortisol levels observed in shift workers (Lindholm et al., 2012). Cortisol is a hormone involved in regulating metabolism, stress response, inflammatory response, and immune function. An alteration in cortisol levels can be detrimental to the functions of the nervous, immune, respiratory, and cardiovascular systems, among others (Oakley and Cidlowski, 2013). In normal sleep, cortisol has very low levels at the beginning of sleep and maximal values at awakening in the morning. However, a circadian mismatch similar to that due to shift work leads to a complete reversal of the cortisol profile, i.e., an increase in cortisol at the beginning of sleep (Scheer et al., 2009). In addition, shift work induces physiological stress due to altered circadian rhythm and decreased sleep quality. Consequently, stress is another factor that can alter cortisol levels in the long term, increasing the risk of cardiovascular disease (Anjum et al., 2011).

The IMF4 could be associated with very slow sympathetic activity because, similar to VLF, it contains frequencies < 0.04 Hz. Previous studies suggest that VLF are related to slow mechanisms of sympathetic function, such as the renin-angiotensin system activity, the mechanism of thermoregulation or metabolism over HRV (Arslan et al., 2019). Shift workers present an increase in scaling exponents at short scales (τ < 128 s), suggesting an alteration in the dynamics of the VLF of the IBI signal. This alteration could be caused by circadian rhythm dysregulation, because VLF may be associated with this phenomenon (Arslan et al., 2019).

Furthermore, a significant decrease in *MFI*(τ) of IBI signal was observed at scales near 256 s in shift workers compared to non-shift workers. This decrease in *MFI*(τ) could be associated with changes in the circadian rhythm of shift workers. Previous studies have shown that the circadian rhythm can affect the self-similarity properties of heartbeats (Hu et al., 2004), particularly between 128 and 256 s (Castiglioni et al., 2020). Castiglioni et al. (2020) studied the dynamics of α(*q*, τ) in cardiovascular series during day and night. They found a decrease in α(*q*, τ) during the night compared to the day, within the range of scales from 128 to 256 s. Similar to the findings of Castiglioni et al. (2020), non-shift workers showed a decrease in α(*q*, τ), although it was more evident at *q*> 0. In addition, shift workers demonstrated a narrower α(*q*, τ) range compared to non-shift workers, mirroring the α(*q*, τ) dynamics observed throughout the day. Therefore, it could be suggested that the impact of circadian mismatch on various physiological processes could lead to a reduction in the α(*q*, τ) variability. In addition, the multifractality quantification results for IMF1–IMF3 indicate higher variability in α(*q*, τ) between 29 ≤ τ ≤ 80 along *q*. Also, a decrease in the variability of α(*q*, τ) of IMF1 and IMF3 at large scales in shift workers suggests a tendency toward monofractal behavior.

Moreover, similar to Castiglioni et al. (2011, 2018), our results suggest that under normal conditions, sympathetic modulation is characterized by Brownian motion components at short scales. On the other hand, vagal modulation presents components that tend to a behavior closer to white Gaussian noise.

The altered sleep macrostructure resulting from night shifts could be another factor influencing the self-similarity properties of the IBI signal. Shift workers experience a decrease in the total sleep duration and changes in the duration of rapid eye movement (REM) sleep (Vogel et al., 2012; Zimberg et al., 2023), and non-REM sleep (Chung et al., 2009; Vogel et al., 2012). Moreover, REM sleep is characterized by greater sympathetic modulation and higher long-term scaling exponents of DFA compared to non-REM sleep (Penzel et al., 2003). Therefore, sleep macrostructure could also contribute to the observed changes in the IBI signal scaling exponents of shift workers. Future studies should explore the multifractal-multiscale properties of the IBI signal in shift workers throughout the sleep cycles, considering altered sleep macrostructure due to shift work.

Finally, it is important to comment the limitations of this study. The first limitation is the small number of recordings due to the cost involved by the polysomnography in the acquisitions. Furthermore, even though it has been demonstrated that IBI signals extracted from BCG or ECG offer similar results in HRV analysis, there is a possibility of differences observed in MMF-DFA analysis due to the method of cardiac activity acquisition. However, with the use of systems similar to PBS, rings or smart watches able to acquire IBI signal, the recordings could be performed in a simple and economical way (Kortelainen et al., 2010; Guerrero et al., 2015). The second limitation is the lack of databases with similar characteristics to perform the study, giving that in our study shift workers and non-shift workers women are not matched by age, which could affect the results since HRV is partially age-dependent (Agelink et al., 2001). Moreover, this analysis did not evaluate the impact of shift work on HRV in male shift workers, which is proposed as a future study to enhance our understanding of how gender may influence HRV responses to shift work. Note that these results are preliminary and cannot be used to follow clinical decisions, since the results need to be validated in a larger population.

## 5 Conclusions

The evaluation of the multiscale-multifractal properties of cardiac function in female shift and non-shift workers was presented, considering the decomposition of the IBI signal into high and low frequency components. The main findings are that both groups exhibit multifractality in their IMFs, mainly at smaller scales. Additionally, cardiovascular stress experienced by shift workers led to an alteration of the scaling exponents of the IBI signal components, particularly at short scales, and considering positive and negative moments. Also, the variability of scaling exponents in IMF1 and IMF3 of shift workers decreased at larger scales despite exhibiting higher variability at small scales. The changes in multiscale-multifractal properties of HRV components are associated with various physiological processes that affect cardiac activity, such as the misaligned circadian cycle due to shift work. Additionally, to our knowledge, this is the first study to evaluate the fractal structure of the IBI signal components separately during sleep for shift workers. The methodology has allowed us to emphasize the contribution of both high and low frequencies of HRV in the overall multiscale-multifractal structure. It was also possible to evaluate sympathetic modulation by separating it from HRV activity where there is an influence of respiration, since the 0.15–0.4 Hz interval corresponds to HRV related to the respiratory cycle. In addition, this methodology identified changes in HRV fractal properties during sleep due to cardiovascular stress experienced by shift work which could be used as clinical indicator for cardiovascular diagnosis.

## Data availability statement

Publicly available datasets were analyzed in this study. This data can be found at: PhysioNet repository (https://physionet.org/content/capslpdb/1.0.0/). However, data of female shift workers are available upon reasonable request from the authors.

## Ethics statement

The studies involving humans were approved by Finnish Institute of Occupational Health. The studies were conducted in accordance with the local legislation and institutional requirements. The participants provided their written informed consent to participate in this study.

## Author contributions

RD-A: Methodology, Software, Writing—original draft. GD-M: Writing—review & editing, Conceptualization, Formal analysis, Methodology. AB: Writing—review & editing, Funding acquisition. JK: Writing—review & editing. SC: Writing—review & editing. JJ-C: Writing—review & editing. MM: Conceptualization, Formal analysis, Funding acquisition, Methodology, Writing—review & editing.
